# Surgically Treated pT2aN0M0 (Stage IB) Non-Small Cell Lung Cancer: A 20-Year Single-Center Retrospective Study

**DOI:** 10.3390/jcm12052081

**Published:** 2023-03-06

**Authors:** Monica Casiraghi, Francesco Petrella, Claudia Bardoni, Shehab Mohamed, Giulia Sedda, Juliana Guarize, Antonio Passaro, Filippo De Marinis, Patrick Maisonneuve, Lorenzo Spaggiari

**Affiliations:** 1 Department of Thoracic Surgery, IEO, European Institute of Oncology IRCCS, 20141 Milan, Italy; 2 Department of Oncology and Hemato-Oncology, University of Milan, 20141 Milan, Italy; 3 Division of Oncology, IEO, European Institute of Oncology IRCCS, 20141 Milan, Italy; 4 Division of Epidemiology and Biostatistics, IEO, European Institute of Oncology IRCCS, 20141 Milan, Italy

**Keywords:** stage IB, non-small cell lung cancer, T2aN0M0, surgery

## Abstract

**Introduction** The suitability of adjuvant therapy (AT) in patients with stage IB non-small cell lung cancer (NSCLC) is still under debate considering the cost–benefit ratio between improvement in survival and side effects. We retrospectively evaluated survival and incidence of recurrence in radically resected stage IB NSCLC, to determine whether AT could significantly improve prognosis. **Methods** Between 1998 and 2020, 4692 consecutive patients underwent lobectomy and systematic lymphadenectomy for NSCLC. Two hundred nineteen patients were pathological T2aN0M0 (>3 and ≤4 cm) NSCLC 8th TNM. None received preoperative or AT. Overall survival (OS), cancer specific survival (CSS) and the cumulative incidence of relapse were plotted and log-rank or Gray’s tests were used to assess the difference in outcome between groups. **Results** The most frequent histology was adenocarcinoma (66.7%). Median OS was 146 months. The 5-, 10-, and 15-year OS rates were 79%, 60%, and 47%, whereas the 5-, 10-, and 15-year CSS were 88%, 85%, and 83%, respectively. OS was significantly related to age (*p* < 0.001) and cardiovascular comorbidities (*p* = 0.04), whereas number of LNs removed was an independent prognostic factor of CSS (*p* = 0.02). Cumulative incidence of relapse at 5-, 10-, and 15-year were 23%, 31%, and 32%, respectively, and significantly related to the number of LNs removed (*p* = 0.01). Patients with more than 20 LNs removed and clinical stage I had a significantly lower relapse (*p* = 0.02). **Conclusions** Excellent CSS, up to 83% at 15-year, and relatively low risk of recurrence for stage IB NSCLC (8th TNM) patients suggested that AT for those patients could be reserved only for very selected high-risk cases.

## 1. Introduction

Lung cancer is still one of the leading causes of death worldwide, being the second most commonly diagnosed cancer after female breast cancer. In the last decades, owing to the wide diffusion of lung cancer screening programs, the vast majority of early-stage NSCLC cases, in particular of the adenocarcinoma type, were readily detected [1,2]. Nevertheless, the 5-year overall survival (OS) of patients with radically resected stage I NSCLC is still considerably heterogeneous, varying from 90% for stage IA1 to 73% for stage IB [3], with a relatively high propensity for recurrence.

Whereas it is widely recognized that NSCLC patients with lymph node involvement have poor prognosis and that their survival could improve with adjuvant treatment (AT) [4,5], the appropriateness of administering AT to stage IB patients (T3-4cmN0M0, T2CentrN0M0, and T2ViscPlN0M0) is still under debate as the cost–benefit ratio between survival rate improvement and side effects are being evaluated.

Retrospective, single-center studies on the topic have given controversial and inconclusive results [6,7,8,9,10,11,12,13], and raised many doubts suggesting discordant recommendations [14,15,16], mostly related to the changes made to the definition of stage IB and IIA NSCLC in the 8th edition of the tumor, node, metastasis (TNM) staging system. Indeed, in the 8th TNM staging system, T2 has been split into T2a (<3–4 cm), the real stage IB, and T2b (>4–5 cm), with T2bN0M0 classed as IIA. This has had an unprecedented effect on the population eligible to receive AT, and has created confusion in interpreting data from studies, which still consider lesions greater than 4 cm as stage IB.

The only multicenter randomized clinical trial (CALGB 9633) specifically designed for stage IB NSCLC patients showed that AT had failed to improve survival except in patients with tumors larger than 4 cm [17]. Thus, the current National Comprehensive Cancer Network (NCCN) guidelines recommend postoperative chemotherapy only for T2bN0M0 NSCLC patients with a tumor ≥4 cm in size (stage IIA according to the 8th TNM), while currently T2aN0M0 patients (stage IB based on the 8th TNM) are usually tailored-treated following the oncologist’s recommendations, unless in case of a high-risk of recurrence.

The aim of this study is to retrospectively analyze radically resected T2aN0M0 (stage IB) NSCLC patients to evaluate their survival and the incidence of recurrence, and to determine whether AT could improve their prognosis.

## 2. Materials and Methods

The study was conducted according to the guidelines of the Declaration of Helsinki; the Ethics Committee of our Institution waived the need for ethics approval and the need to obtain consent for the collection, analysis, and publication of the retrospectively obtained and anonymized data for this non-interventional study. Written informed consent was obtained from all patients at the time of surgery. All data underlying this article are available in the article and in its online Supplementary Materials. This study was reported based on the Strengthening the Reporting of Observational Studies in Epidemiology (STROBE) checklist for cross-sectional studies.

We selected patients who, according to the 8th TNM edition, were affected by pathological T2a NSCLC (tumor size >3 and ≤4 cm) and that showed no pathological lymph node involvement. We excluded from the study all patients with one or more of the following characteristics: prior treatments or history of cancer within the previous 5 years, histology other than NSCLC, incomplete preoperative staging, incomplete lymphadenectomy, anatomical resection other than lobectomy, incomplete resection (R1 or R2 resection), preoperative or adjuvant treatments such as chemotherapy, biological therapy, immunotherapy, or radiotherapy.

For all patients, preoperative staging was based on total body computed tomography scan (CT-scan), positron emission tomography (PET) with fluorodeoxyglucose (FDG), cardiological examination, and pulmonary function test followed by anesthesia evaluation. Whenever possible, suspected mediastinal lymph node involvement was verified with either endobronchial ultrasound-guided transbronchial needle aspiration (EBUS-TBNA) or mediastinoscopy [18]. Staging and functional exams were always performed in the 5 weeks before surgery. During the multidisciplinary meetings, thoracic surgeons and oncologists confirmed patient resectability and medical treatment plans.

All patients underwent pulmonary lobectomy and radical lymphadenectomy. In all patients, systematic lymph node dissection was performed according to the classification of the American Thoracic Society by removing all lymphatic tissue from stations 2R, 4R, 7, and 10R for right-sided tumors and from stations 5, 6, 7, and 10 L for left-sided tumors. A complete pathologically resection was defined R0, microscopic residual disease at pathology was defined R1, whereas a macroscopically incomplete resection was defined R2. Postoperative complications were defined according to the Clavien–Dindo classification [19].

Patients received a physical follow-up, chest X-ray, and blood tests at 1 month after surgery; then, they received a physical examination plus a chest and upper abdomen CT-scan every 4 months for the first 2 years, every 6 months for the following 3 years, and annually after 5 years from surgery.

Recurrence at the site of surgery (hilar/mediastinal region or lung parenchyma close to the previous resection), in the chest cavity (ipsilateral and contralateral such as new pulmonary nodules or pleural diffusion) and distant metastasis were recorded and classified as local, regional, and distant, respectively. We did not consider recurrence but a second primary tumor when a distinct pulmonary malignancy displayed different histology or different morphology by comprehensive histologic assessment, and/or it was diagnosed 2 or more years after the first primary lung cancer, in the absence of nodal or systemic metastases. In the case of adenocarcinoma histology, considering the retrospective nature of the study and the impossibility to have a molecular mutational analysis performed on any paired primary tumor and suspected recurrent tumor to distinguish true recurrence from second primary tumor, the second event was considered recurrence.

### Statistical Analysis

Overall survival (OS) and cancer-specific survival (CSS) were calculated using the date of surgery and the date of either last contact or death. Relapse was defined from the date of surgery to the date of any relapse (local, regional, or distant), last contact, or death. OS and CSS were plotted using the Kaplan–Meier method, and the log-rank test was used to assess outcome differences between groups. For time to recurrence, we considered death as competing risk and use cumulative incidence plots with the Fine and Gray test. Analyses were performed with SAS software version 9.4 (SAS Institute, Cary, NC, USA). All P values were two-sided; *p* < 0.05 was considered statistically significant.

## 3. Results

We retrospectively evaluated 4692 consecutive patients who, between January 1998 and December 2020, underwent lobectomy and systematic lymphadenectomy for NSCLC at the European Institute of Oncology (IEO) in Milan, Italy. Among them, 219 were pathological T2aN0M0 (stage IB) NSCLC patients whose characteristics and tumor details are shown in Table 1: they were mostly men (68.9%); aged 41–89 years; with at least one comorbidity (88.1%); 8 patients out of 219 were clinical stage IA1 (3.6%), 35 IA2 (16%), 59 IA3 (26.9%), 71 IB (32.4%), 40 IIA (18.3%), and 6 IIB (2.7%).

Open surgery was performed in 156 patients (71.2%), whereas 56 (25.6%) were surgically approached in RATS, and 14 (6.4%) in VATS. Three patients were converted to open surgery from either VATS (n = 2) or RATS (n = 1), due to adhesions.

All patients underwent lobectomy and systematic lymphadenectomy; 7 patients out of 219 (3.2%) underwent sleeve resection (6 bronchial and 1 bronchial and vascular). All 219 patients had a pathological radical resection (R0).

The pathological size of the primary tumor was 30–35 mm in 142 patients (64.8%), and 36–40 mm in 77 (35.2%).

Adenocarcinoma was the most frequently found histologic type (n = 146; 66.7%), followed by squamous carcinoma (n = 61; 27.9%), and adeno-squamous carcinoma (n = 10; 4.6%). The median total number of lymph nodes removed was 16 (range 4–40). Visceral pleural infiltration was evident in 78 cases (35.6%).

Postoperative complications and outcomes are listed in Supplementary Table S1. Major complications (Clavien–Dindo 3 and 4) were observed in 9 patients (4.1%): 1 hemothorax and 1 chylothorax, both requiring surgical intervention; 1 bronchopleural fistula, treated with a completion pneumonectomy; 1 acute cholecystitis, requiring cholecystectomy; 1 empyema treated with chest tube and antibiotic therapy; 2 bronchial toilettes, and 2 acute respiratory distress syndrome (ARDS) requiring intensive care unit (ICU). Minor complications (Clavien–Dindo 1 and 2) occurred in 58 patients (26.48%), mostly atrial fibrillation (n = 27; 12.32%) and pleural air leak (n = 17; 7.76%).

The mean hospital stay for all patients was 6 days (range, 3–44 days). No intraoperative mortality was observed.

**Figure 1:**
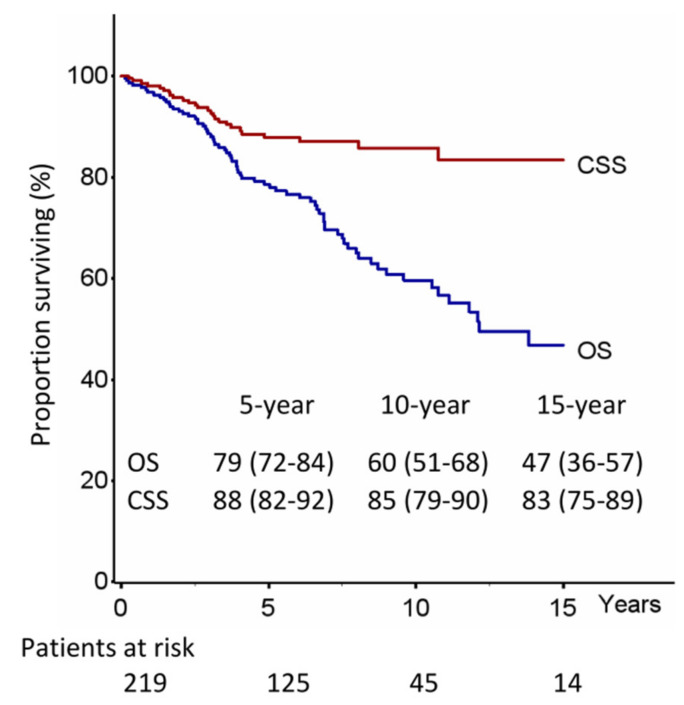
Overall survival (OS) and lung cancer specific survival (CSS).

The median OS was 146 months, with 5-, 10-, and 15-year OS rates of 79%, 60%, and 47%, whereas the CSS at 5-, 10-, and 15-year were 88%, 85%, and 83%, respectively (Figure 1).

**Figure 2:**
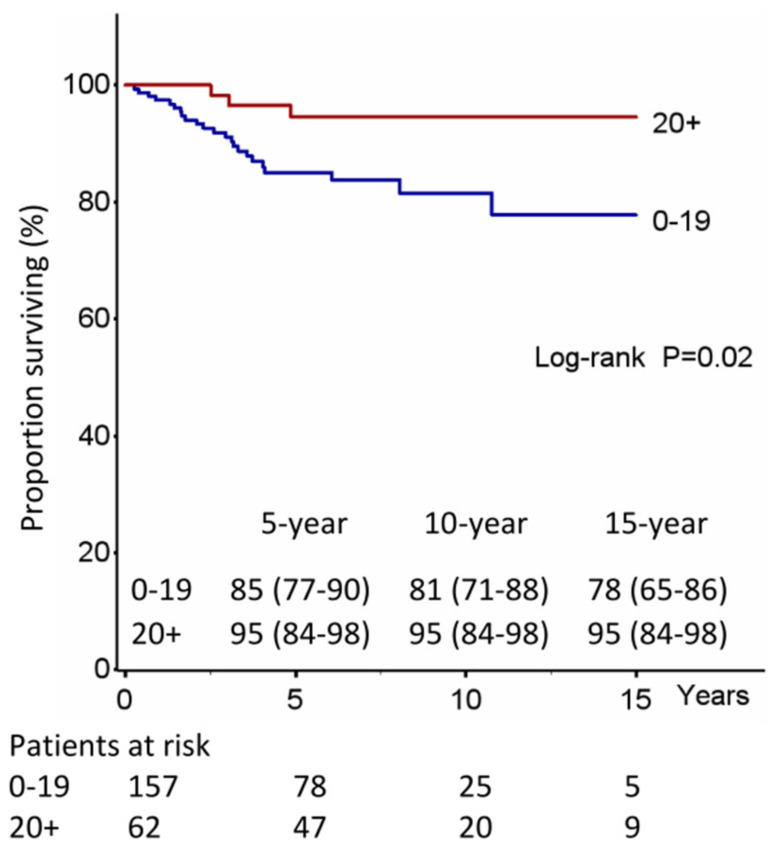
Lung cancer specific survival (CSS) by number of LN removed.

Whereas OS was significantly related to age (*p* < 0.001) and to cardiovascular comorbidities (*p* = 0.04), the number of lymph nodes removed was an independent prognostic factor of CSS (*p* = 0.02) (Figure 2 and Table 2).

A total of 55 (25.1%) patients developed recurrences of the disease (Table S1 and Figure 1): 15 (6.8%) had local relapse, 18 (8.2%) regional recurrences, and 26 (11.9%) distant metastases (2 patients had simultaneous local and distant relapse and 1 patient simultaneous local, regional and distant relapse).

Of the 55 patients with recurrences, 38 (73.1%) developed adenocarcinoma, with 3 of them (5.7%) harboring EGFR mutations. The recurrences were mostly treated with radiotherapy alone (n = 21, 40.4%), followed by platinum-based chemotherapy (n = 18, 34.6%). The three patients with EGFR mutations were treated with Osimertinib. Combined treatments (platinum-based chemotherapy with either radiotherapy or immunotherapy) were indicated in eight patients (15.4%) who developed distant metastasis. Instead, one patient who developed a single metastasis in the contralateral lung underwent surgical metastasectomy.

**Figure 3:**
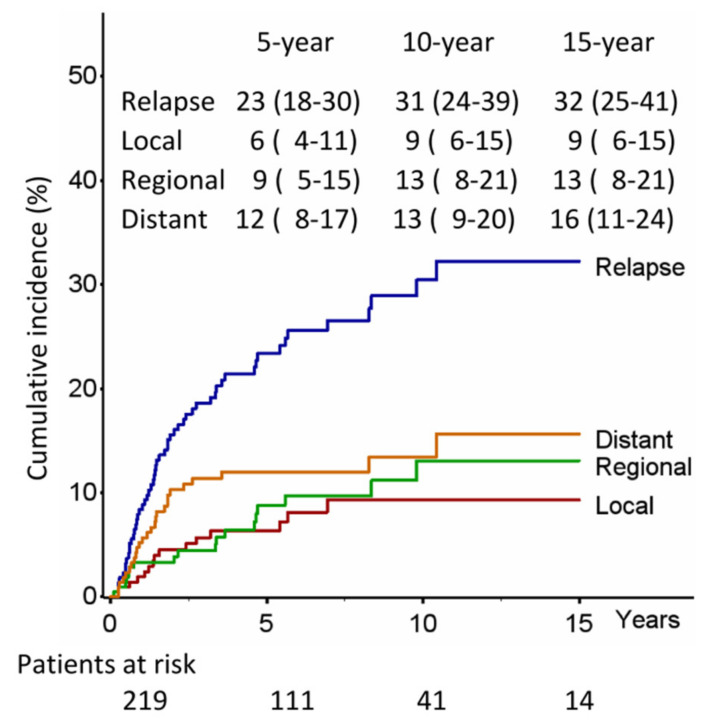
Lung cancer recurrence.

**Figure 4:**
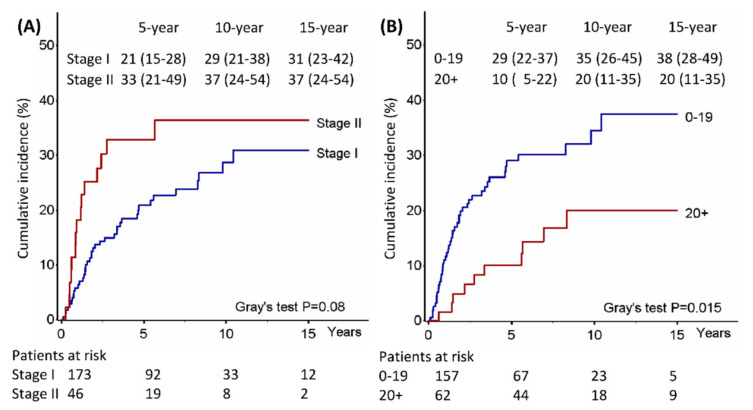
Cumulative incidence of relapse by clinical stage (**A**) or number of resected nodes (**B**).

The cumulative incidence of relapse at 5-, 10-, and 15-year was 23%, 31%, and 32%, respectively (Figure 3), and related to clinical stage (*p* = 0.08) and number of LNs removed (*p* = 0.01) (Figure 4 and Table 2); patients with more than 20 LNs removed and clinical stage I had a significantly lower probability of relapse than patients with less than 20 LNs removed or clinical stage II (*p* = 0.02) (Figure S1), whereas the CSS was significantly related only to the number of LNs removed (*p* = 0.02).

## 4. Discussion

When taking as reference the 8th edition of the TNM staging system, the 5-year OS rate for NSCLC patients is about 73% for stage IB cases and 80–90% for stage IA cases. Thus, in the last decade, oncologists and surgeons have often discussed the opportunity to propose to stage IB patients a postoperative treatment aimed to improve survival and to reduce recurrences. The latest guidelines include recommendations for postoperative treatment in resected stage II–IIIA and in selected stage IB NSCLC patients, usually associated with poor prognosis and disease recurrence [15,16]. However, mostly due to the incorrect staging of these patients and the confusion in interpreting data that still consider lesions larger than 4 cm as stage IB, based on the 7th edition of the TNM staging system, there are still many doubts on the benefits of postoperative treatment on the survival of these patients. Furthermore, rates of relapse after surgery still remain highly dependent on disease stages (45% for stage IB disease; 62% for stage II disease; 76% for stage III disease), notwithstanding the use of adjuvant chemotherapy [20].

The Cancer and Leukemia Group B 9633 study showed that adjuvant chemotherapy improved survival in patients with tumors larger than 4 cm in size, but not in those with tumors smaller than 4 cm (HR, 1.12; 90% CI, 0.75 to 1.07; *p* = 0.32) [21]. However, when reanalyzing the patient population based on the 8th TNM edition, only a few stage IB NSCLC patients were included without considering possible high-risk factors. Even the ANITA trial, which showed the benefits of adjuvant chemotherapy mainly in patients with lymph node metastasis, unfortunately considered only stage IB cases as defined in the 7th TNM edition.

In 2021, Wang [22] performed the largest and most recent systematic review and meta-analysis (including 12 eligible studies for a total of 15,678 patients) and showed that AT might provide survival benefits in patients with stage IB NSCLC, independently from histology or regimens. This study also addressed the issue of whether AT should be administered in the case of malignancies <4 cm: only 7 out of the 12 studies taken into consideration showed better OS and gave support to the routine use of AT for stage IB NSCLC patients. This number decreased to only two in the case of adenocarcinoma histology [22].

In our study, we analyzed selected T2aN0M0 (size 3–4 cm, stage IB in the 8th TNM edition) NSCLC patients surgically treated without AT and found that the 5-year OS was 79%, and the 5-year CSS was 88%, staying at up to 83% for as long as 15 years. In comparison to the available literature, we showed a higher survival rate and the importance of correct staging and data interpretation. Real stage IB cases, as defined in the 8th TNM, are indeed smaller than 4 cm in size and have better survival outcomes than those similarly staged in the previous edition of the staging system. Therefore, AT should not be prescribed a priori to improve survival in patients with an already good prognosis, and used only in carefully selected high-risk cases.

Several are the factors that are considered to be high-risk, among them poor tumor differentiation, vascular invasion, wedge resection, visceral pleural involvement, and incomplete lymph node dissection [14], but a clear, clinically applicable risk-stratification model for the identification of stage IB NSCLC is not yet available. It is unknown whether there is a risk difference between them and the efficacy of adjuvant chemotherapy in those high-risk patients, creating confusion in clinical practice, as the use of adjuvant chemotherapy relies on the clinician’s judgment [23,24].

In a recent study, Zhai et al. proposed a clinical risk score (CRS) based on the patients’ detailed risk factors, predicted the prognosis of patients with stage IB-IIA NSCLC, and found a significant association between adjuvant chemotherapy and the prognosis of patients with stage IB-IIA NSCLC, particularly those with a high clinical risk score and non-squamous cell histology. Unfortunately, they did not manage to make a clear distinction between stage IB and IIA cases [25].

In 2022, Choi et al. published a retrospective multicenter study including 285 stage IB NSCLC patients with high-risk factors as defined by the 8th TNM edition. They showed that adjuvant chemotherapy was beneficial in this cohort of patients and that it significantly reduced their risk of recurrence and mortality. In the multivariate analysis, the adjuvant chemotherapy group had a significantly lower recurrence rate and risk of mortality than the control group, in particular in patients with high-risk factors such as visceral pleural involvement or vascular invasion [26].

In the phase 3 ADAURA study (NCT02511106) [27], Osimertinib was found to have a clinically meaningful effect on disease-free survival in patients with resected stages IB to IIIA EGFR-mutated (EGFRm) NSCLC, irrespective of whether patients had previously received chemotherapy or not. However, only 26% of patients with stage IB disease received adjuvant chemotherapy compared to 76% of II to IIIA stage cases. Moreover, also in the ADAURA study, stage IB cases were classified as such according to the 7th TNM edition, and were therefore actual stage IIA cases.

In our study, the incidence of relapse rates at 5-, 10-, and 15-year were 23%, 31%, and 32%, respectively, and significantly associated with the number of lymph nodes removed (*p* = 0.01); moreover, in a subgroup analysis, patients with more than 20 lymph nodes removed and clinical stage I had a probability of relapse significantly lower than patients with less than 20 lymph nodes removed or clinical stage II (*p* = 0.02) (Figure 4). Additionally, the number of lymph nodes removed was also an independent prognostic factor of CSS (*p* = 0.02), making it the only high-risk factor related to survival, unlike size and visceral pleura which, on the other hand, did not correlate with either survival or risk of recurrence.

This was an important finding as the high survival rate we observed in our study could be explained with a proper postoperative staging resulting from the radical and systematic lymphadenectomy we routinely performed. The lower survival rates found by others could be due to cases being wrongly staged as IB following incomplete removal of lymph nodes.

Our study presents a few limitations. Firstly, its retrospective nature placed limits and introduced biases on all the variables included in the analysis. Secondly, it did not include a control group undergoing AT, as we do not routinely administer AT to stage IB NSCLC, except for a highly selected cohort of high-risk patients. As the use of Osimertinib was allowed only recently in cases carrying EGFR mutations, only three patients were included in this study, too few to make any considerations. The main strength of our study is in relying on data from a single center, with uniform indications and patients’ selection over the years. In addition, the data presented herein were collected by a high-volume referral center for more than twenty years, so that noteworthy conclusions can be drawn from them.

## 5. Conclusions

Based on the excellent CSS (up to 86% at 15-year, with a relatively low risk of recurrence) observed for the stage IB (8th TNM) patients in our study, AT could be prescribed only to selected high-risk factors cases. 

## Figures and Tables

**Table 1:** Characteristics of 219 patients with stage IB (pT2aN0M0) NSCLC operated at the IEO during 1998–2020, who did not receive neoadjuvant or adjuvant therapy for primary cancer.

Patients’ Characteristics	N (%)	Surgical Characteristics	N (%)	Tumor Characteristics	N (%)
Age, years, Median [range]	67 [40–88]	Surgical approach		Histology	
<60	36 (16.4)	Open lobectomy	149 (68.0)	Adenocarcinoma	146 (66.7)
60–69	100 (45.7)	RATS	56 (25.6)	Squamous	61 (27.9)
70+	83 (37.9)	VATS	14 (6.4)	Adeno-squamous	10 ( 4.6)
Sex		Conversion	3 (1.4)	NSCLC	2 ( 0.9)
Men	151 (68.9)	Sleeve	7 (3.2)	Tumor size	
Women	68 (31.1)	Nodule site		30–35mm	142 (64.8)
Comorbidities		Upper lobe	146 (66.7)	36–40mm	77 (35.2)
Other Cardiac	125 (57.1)	Middle lobe	6 (2.7)	Tumor grade	
Ischemic heart disease	21 (9.6)	Lower lobe	67 (30.6)	G1	18 ( 8.2)
Hypertension	87 (39.7)	Nodule side		G2	78 (35.6)
Pulmonary	33 (15.1)	Left	132 (60.3)	G3	115 (52.5)
COPD	25 (11.4)	Right	87 (39.7)	Missing	8 ( 3.7)
Other	176 (80.4)	Lymph nodes removed		Visceral pleura infiltration	
Clinical Stage		Total, median [range]	16 [4–40]	Absent	141 (64.4)
I	173 (79.0)	N1, median [range]	6 [1–28]	Present	78 (35.6)
II	46 (21.0)	N2, median [range]	8 [0–25]		

COPD: chronic obstructive pulmonary disease; RATS: robotic-assisted thoracic surgery; VATS: video-assisted thoracic surgery; NSCLC: non-small cell lung cancer; VP: visceral pleura.

**Table 2:** Factors associated with tumor relapse, cancer specific survival (CSS), and overall survival (OS).

Characteristics		Tumor Relapse			Cancer Specific Survival (CSS)			Overall Survival (OS)		
	N (%)	Events	HR (95% CI)	Gray’s Test	Deaths	HR (95% CI)	Log-Rank	Deaths	HR (95% CI)	Log-Rank
**Total**	**219**	55						75		
Age										
<60	36 (16.4)	6	1.00		3	1.00		5	1.00	
60–69	100 (45.7)	26	1.63 (0.68–3.90)		11	1.44 (0.40–4.16)		30	2.67 (1.03–6.91)	
70+	83 (37.9)	23	1.80 (0.74–4.38)	0.41	12	2.04 (0.57–7.31)	0.47	40	**5.99 (2.33–15.4)**	**<0.0001**
Sex										
Men	151 (68.9)	37	1.00		15	1.00		54	1.00	
Women	68 (31.1)	18	1.10 (0.63–1.93)	0.73	11	1.65 (0.76–3.60)	0.20	21	0.83 (0.50–1.40)	0.49
Comorbidities										
Cardiac	125 (57.1)	32	1.15 (0.68–1.94)	0.60	15	1.20 (0.55–2.63)	0.65	45	**1.64 (1.02–2.63)**	**0.04**
Myocardial infarction	21 (9.6)	6	1.06 (0.47–2.37)	0.90	2	0.80 (0.19–3.39)	0.76	11	1.85 (0.97–3.52)	0.06
Hypertension	87 (39.7)	22	1.07 (0.63–1.84)	0.77	9	0.93 (0.41–2.09)	0.86	28	1.27 (0.79–2.04)	0.32
Pulmonary	33 (15.1)	7	0.80 (0.37–1.76)	0.59	4	1.00 (0.34–2.99)	0.99	12	1.12 (0.60–2.07)	0.73
COPD	25 (11.4)	7	1.07 (0.49–2.37)	0.86	4	1.30 (0.45–3.77)	0.63	11	1.26 (0.66–2.39)	0.48
Other	176 (80.4)	50	**3.06 (1.2–7.67)**	**0.01**	21	1.24 (0.46–3.33)	0.67	56	1.27 (0.74–2.19)	0.39
Clinical Stage										
I	173 (79.0)	40	1.00		21	1.00		60	1.00	
II	46 (21.0)	15	1.68 (0.92–3.09)	0.08	5	1.00 (0.38–2.65)	0.99	15	1.14 (0.64–2.01)	0.66
Surgical approach										
Open lobectomy	149 (68.0)	39	1.00		21	1.00		61	1.00	
RATS	56 (25.6)	13	1.09 (0.58–2.05)		4	0.45 (0.23–1.94)		10	0.80 (0.40–1.57)	
VATS	14 (6.4)	3	0.78 (0.27–2.29)	0.86	1	0.50 (0.07–3.76)	0.62	4	0.85 (0.31–2.36)	0.78
Conversion	3 (1.4)	0	-	0.30	0	-	0.50	2	1.43 (0.35–5.87)	0.61
Sleeve	7 (3.2)	1	0.47 (0.06–3.56)	0.43	1	0.99 (0.14–7.38)	0.99	3	0.79 (0.25–2.51)	0.68
Nodule site										
Upper lobe	146 (66.7)	34	1.00		17	1.00		49	1.00	
Middle lobe	6 (2.7)	1	1.30 (0.75–2.24)		0	-		1	1.13 (0.69–1.83)	
Lower lobe	67 (30.6)	20	0.70 (0.10–4.78)	0.58	9	1.13 (0.51–2.54)	0.65	25	0.42 (0.06–3.06)	0.58
Nodule side										
Left	132 (60.3)	20	1.00		13	1.00		30	1.00	
Right	87 (39.7)	35	1.13 (0.65–1.96)	0.66	13	0.65 (0.30–1.40)	0.26	45	1.06 (0.67–1.70)	0.80
Lymph nodes removed										
≥20	62 (28.3)	10	1.00		3	1.00		24	1.00	
<20	157 (71.7)	45	**2.27 (1.17–4.39)**	**0.01**	23	**3.79 (1.13–12.6)**	0.02	51	1.25 (0.77–2.05)	0.37
Histology										
Adenocarcinoma	146 (66.7)	40	1.00		16	1.00		39	1.00	
Squamous	61 (27.9)	12	0.64 (0.33–1.24)		7	0.96 (0.40–2.34)		31	1.48 (0.91–2.38)	
Adeno-squamous	10 (4.6)	3	1.12 (0.34–3.63)		3	2.87 (0.83–9.88)		4	1.51 (0.54–4.24)	
NSCLC	2 (0.9)	0	-	0.43	0	-	0.32	1	2.29 (0.31–16.8)	0.36
Tumor size										
30–35 mm	142 (64.8)	39	1.00		15	1.00		48	1.00	
36–40 mm	77 (35.2)	16	0.76 (0.42–1.36)	0.35	11	1.41 (0.65–3.07)	0.39	27	1.12 (0.70–1.81)	0.63
Tumor grade										
G1	18 (8.2)	3	1.00		2	1.00		5	1.00	
G2	78 (35.6)	22	2.12 (0.68–6.58)		9	1.33 (0.29–6.15)		26	1.78 (0.68–4.66)	
G3	115 (52.5)	28	1.69 (0.55–5.21)	0.39	14	1.30 (0.30–5.72)	0.93	42	1.77 (0.70–4.48)	0.46
Missing	8 (3.7)	2	-		1					
Visceral pleura infiltration										
Absent	141 (64,4)	34	1.00		19	1.00		53	1.00	
Present	78 (35.6)	21	1.11 (0.65–1.89)	0.71	7	0.66 (0.29–1.57)	0.34	22	0.80 (0.48–1.31)	0.37

CSS: cancer specific survival; OS: overall survival; COPD: chronic obstructive pulmonary disease; RATS: robotic-assisted thoracic surgery; VATS: video-assisted thoracic surgery; NSCLC: non-small cell lung cancer. Bold text indicates a statistically significant difference with a *p*-value less than 0.05.

## Data Availability

Available upon request.

## Supplementary Materials

The following supporting information can be downloaded at: https://www.mdpi.com/article/10.3390/jcm12052081/s1, Table S1: Patients’ outcomes; Figure S1: Relapse according to clinical stage and number of lymph nodes removed.
