# Supplementary material for: Surgically Treated pT2aN0M0 (Stage IB) Non-Small Cell Lung Cancer: A 20-Year Single-Center Retrospective Study
Source: J Clin Med. 2023 Mar 6;12(5):2081. doi: 10.3390/jcm12052081 (PMC10004231; doi:10.3390/jcm12052081)
Supplement: Supplementary file 1 [file jcm-12-02081-s001.zip › Table S1.pdf]

Table S1. Patients' outcomes

PATIENTS' OUTCOMES	N (%)
Need for ICU	48 (21.9)
Hospital days, median [range]	6 [3-44]
Post-operative complications	67 (30.6)
Pulmonary	32 (14.6)
Cardiac	39 (17.8)
Other	12 (5.5)
Minor	57 (26.0)
Major	10 (4.6)
Clavien-Dindo Classification	
1	37 (16.9)
2	21 (9.6)
3a	3 (1.4)
3b	3 (1.4)
4a	3 (1.4)
Relapse*	52 (23.7)
Local	12 (5.5)
Regional	12 (5.5)
Distant	27 (12.3)
Death	75 (34.2)
Dead of disease	24 (11.0)
Dead of other cause	51 (23.3)

*Site of relapse is missing for 1 patient, 3 patients had relapse at multiple sites.

ICU: Intensive Care Unite
