# Supplementary figures and images for: Surgically Treated pT2aN0M0 (Stage IB) Non-Small Cell Lung Cancer: A 20-Year Single-Center Retrospective Study
Source: J Clin Med. 2023 Mar 6;12(5):2081. doi: 10.3390/jcm12052081 (PMC10004231; doi:10.3390/jcm12052081)

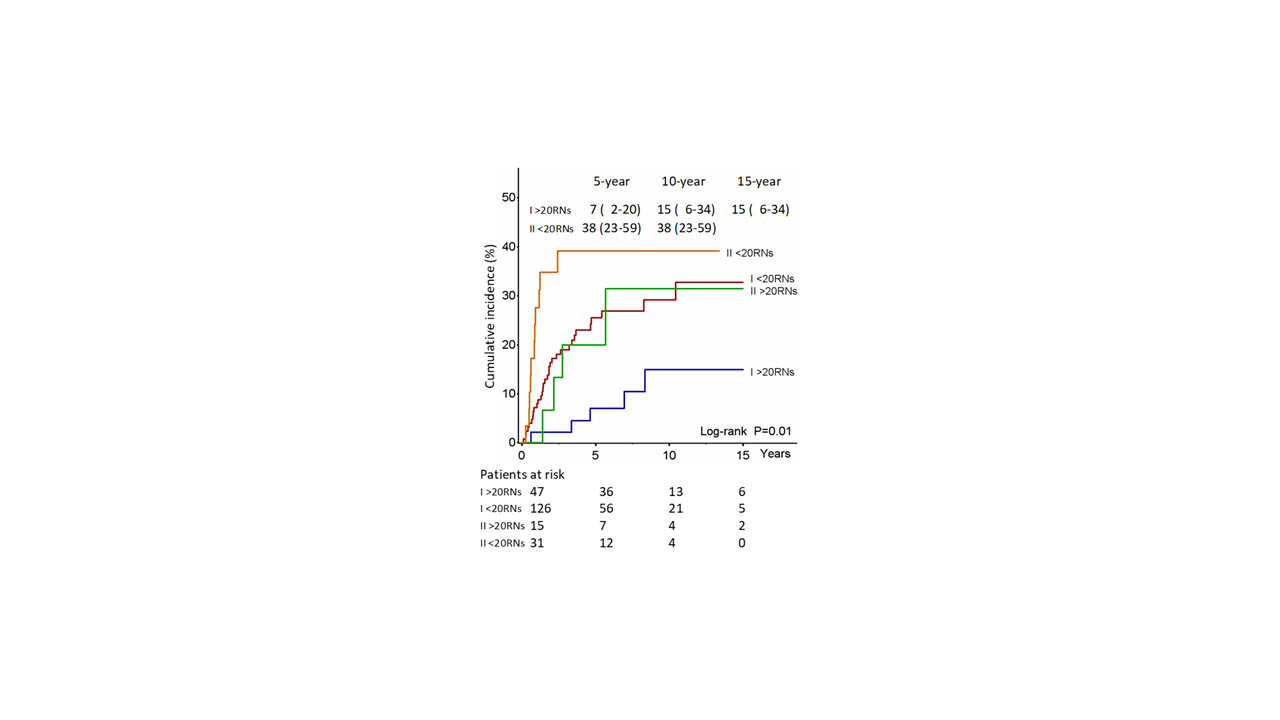

Supplement: Supplementary file 1 [file jcm-12-02081-s001.zip › Figure S1.tif]
